# Adverse childhood experiences, epigenetics and telomere length variation in childhood and beyond: a systematic review of the literature

**DOI:** 10.1007/s00787-019-01329-1

**Published:** 2019-04-09

**Authors:** Jason Lang, Judith McKie, Helen Smith, Angela McLaughlin, Christopher Gillberg, Paul G. Shiels, Helen Minnis

**Affiliations:** 1 Institute of Health and Wellbeing, MVLS, University of Glasgow, Glasgow, UK; 2 NHS Lanarkshire Child and Adolescent Mental Health Services for Learning Disability, Motherwell, UK; 3 NHS Greater Glasgow and Clyde Forensic CAMHS Team, Glasgow, UK; 4 Department of Clinical Psychology, University of Edinburgh, Edinburgh, UK; 5 Gillberg Neuropsychiatry Centre, University of Gothenburg, Gothenburg, Sweden; 6 Institute of Cancer Sciences, MVLS, University of Glasgow, Glasgow, UK

**Keywords:** Adverse childhood experiences, Epigenetics, Biomarker, Child abuse and neglect

## Abstract

A systematic review following PRISMA guidelines was conducted to answer the question: What epigenetic, telomeric and associated biological changes are associated with exposure to adverse childhood experiences (ACEs) in the under 12s? Using PRISMA guidelines, appropriate databases were searched. 190 papers were returned with 38 articles fully reviewed. Articles were each independently quality rated by two authors using the Crowe Critical Appraisal Tool and data were extracted. Of the 38 articles, 23 were rated as very high quality. Most study participants were adults (*n* = 7769) with *n* = 727 child participants. Only seven of the very/high-quality studies were prospective and involved children. Methylation was the most studied method of epigenetic modification. There is some evidence supporting epigenetic modification of certain markers in participants exposed to ACEs measured in adulthood. Research is lacking on non-coding aspects of the epigenome and on coding aspects other than DNA methylation. There is some evidence of a more powerful effect on telomere length if physical neglect was involved. Much further work is required to model biological and psychological effects of epigenetic changes during childhood using prospective study designs. The effect of ACEs on the cellular ageing process during childhood is inadequately investigated and relies solely on measure of telomere length. Future research suggestions are proposed.

## Introduction

It is broadly accepted that exposure to adverse childhood experiences (ACEs) is associated with a number of detrimental biological, psychological and social aspects of health [1–3]. Early life stress is associated with dysregulation of neurochemical systems, such as the hypothalamic–pituitary–adrenal axis, and this dysregulation can increase vulnerability to anxiety and mood regulation disorders [4, 5]. It is also accepted that exposure to ACEs can predispose to physical illness [6].

The precise mechanisms involved in these alterations have yet to be fully elicited. Research has examined genetic loci involved with immune, stress and neurobiological responses to early-life insults. The concept of “progeria” (accelerated ageing) has been studied by looking at the effects of ACEs on reducing the length of telomeres (the cap at the end of DNA strands which acts to protect the strand) (for examples, see [7–12]). Despite international interest in understanding the link between ACE exposure and epigenetics [13] this picture is incomplete, especially regarding changes occurring during childhood.

The study of the epigenome has opened new areas of knowledge and understanding of environmental factors affecting the normative developmental/ageing process. Epigenetic changes are defined as a set of heritable changes that are not coded for in the underlying DNA sequence. Epigenetic changes provide a way of changing the phenotype (how an organism/cell looks or behaves) without changing the genotype (the underlying DNA). Epigenetic regulation of gene expression is thought to have evolved to allow the body to respond rapidly to environmental changes [14] and is critical for cellular development and maintenance of cellular homeostasis, especially in response to alterations in the environment [15].

Broadly, there are two types of epigenetic modification. “Canonical” modifications involve coding DNA or histones (nuclear protein) and include DNA *methylation* (*hypo- or hyper*-*methylation*), or addition of molecular groups to histones through *acetylation/phosphorylation*, which modify protein functioning, and *ubiquitination/sumoylation* (both of which cause cell death) [14, 15]. Such epigenetic changes coordinate the access of transcriptional machinery to enable or preclude DNA expression thus regulating changes in phenotype, without changes in genotype. “Non-canonical” modifications involve regulatory networks of non-coding RNAs (for example, microRNAs). These integrate canonical features within an overarching epigenetic landscape and may be as important in modifying phenotype as changes to coding DNA and its apparatus.

Both exogenous (environmental) and endogenous (physiological) stimuli lead to changes in the epigenetic landscape that impact on normative developmental and age-related physiological capability.

ACEs could, therefore, alter gene expression, potentially doing so via alterations in the epigenetic landscape of affected cells and tissues in those exposed to childhood adversity with direct implications for future physical and mental health. Most research on ACE-related premature ageing has been in adult populations and there is a critical need to examine the extent to which any changes begin in childhood, the time when changes are likely to be most reversible.

### Aims of the study

Our aim was to systematically review the available literature regarding ACE exposure and epigenetic modification in childhood, to highlight knowledge gaps and propose future research directions. The research question for this systematic review is: What epigenetic, telomeric and associated bio-physiological factors or processes are associated with exposure to ACEs in children aged under 12 years? Given the limited returns in initial scoping searches it was decided to include relevant papers with adult participants retrospectively reporting ACEs exposure which otherwise met the inclusion criteria.

## Methods

### Search characteristics and protocol

Preferred Reporting Items for Systematic Reviews and Meta-Analyses (PRISMA) guidelines were followed.  To access appropriate literature for this review a search strategy was devised and applied to Medline, Psychinfo and Embase databases. The first phase of literature searches was conducted in November 2016, with an update using the same methodology conducted in February 2018.

An initial search was conducted using the key terms *biochemical markers.mp* OR *exp. Biomarkers.* A further search was conducted using the terms *epigenetic.mp* OR *exp epigenomics.* Additional refiners were added to include *epigenetic* AND *marker* OR *modification,* with a further addition of three-word separator using the above terms. A further search was conducted using the terms *telomere.mp* OR *exp telomere shortening* OR exp *telomere* OR *exp telomere homeostasis.*

The search strategy was then expanded to include terms associated with childhood maltreatment and known related disorders including permutations of *exp depressive disorder* OR *exp life change events* OR *exp anxiety disorders*, OR exp stress, psychological OR early adversity. Further permutations including *exp stress disorders, post traumatic* OR *childhood adversity.mp* were added. Permutations of *childhood* AND *adversity* OR *childhood.mp* were then added. Additional searches were performed using *exp Child Health* OR *exp Mother*-*Child Relations* OR *Childcare* OR *exp Child Preschool* OR *exp Parent*-*Child Relations.*

The search was expanded further to include related terms: *exp Child Welfare* OR *exp Psychology, child* OR *exp Child, orphaned* OR *exp Child Abandoned* OR *exp Child, Abandoned* OR *exp Child Behaviour Disorders* OR *Child Reactive Disorders* OR *Child Development* OR *child.mp* OR *exp Child Psychiatry* OR *exp Child Protective Services* OR *exp Battered Child Syndrome* OR *exp Father*-*Child Relations* OR *exp Child Behaviour.*

Further combined key term combinations were searched for using the terms *Biomarker* AND *Abuse* OR *Sexual* OR *physical* OR emotional OR molest* OR *Non Accidental Trauma and separately the terms Epigenetic* AND *Sexual* OR *Physical* OR *emotional* OR molest* OR *Non Accidental Trauma.*

The results of the above searches were combined and limited to (i) English language, (ii) publication date from 2006 and (iii) (initially) children under the age of 12 years. This third limitation was then removed as described above. Duplicate titles were electronically removed.

### Inclusion and exclusion criteria

Inclusion and exclusion criteria were discussed and agreed after initial scoping searches with the authors (JL, PS and HM). Articles were screened for inclusion or exclusion by a single author (JL), according to the inclusion criteria below, using title and abstract review. During the screening process, where there was any uncertainty, inclusion or exclusion was discussed with PS and HM. Articles were included in the final list where

- the paper comprised of original research;
- the paper had epigenetic, telomere or biomarker data as primary or secondary measures;
- the participants were human and had been exposed to ACEs (prospectively or retrospectively reported);
- the full article was accessible for review by the authors.

### Paper rating process

Following title and abstract review for inclusion, remaining papers were read and rated using the Crowe Critical Appraisal Tool (CCAT) (v1.4) (Crowe Critical Appraisal Tool V1.4 https://conchra.com.au/wp-content/uploads/2015/12/CCAT-form-v1.4.pdf) initially by one author (JL). A random sample (*n* = 17) of papers were read and reviewed separately by additional authors (HM and JM) to examine inter-rater reliability of the rating process. All authors were blind to the ratings of fellow authors during independent rating. Where there was a rating discrepancy of greater or equal to five points, this article was discussed between all three rating authors and consensus was reached on the final rating at conference. Four papers of the 38 papers generated were re-rated in this way. Subsequently, all 38 papers were independently rated by two authors. An additional four papers required conference discussion with consensus reached on the final rating. Where the discrepancy was less than five points, (i.e. not requiring conference) the ratings were averaged to achieve a mean score.

Using these ratings, papers were allocated to one of four categories, ranging from very high quality (VHQ) papers (score of 35 or greater), high-quality (HQ) papers (score of 30 or greater), moderate-quality (MQ) papers (score 20 or greater) or low-quality (LQ) papers (score of less than 20). The authors agreed that any papers receiving a score of less than 20 would not be included in the qualitative synthesis.

## Results

**Fig. 1:**
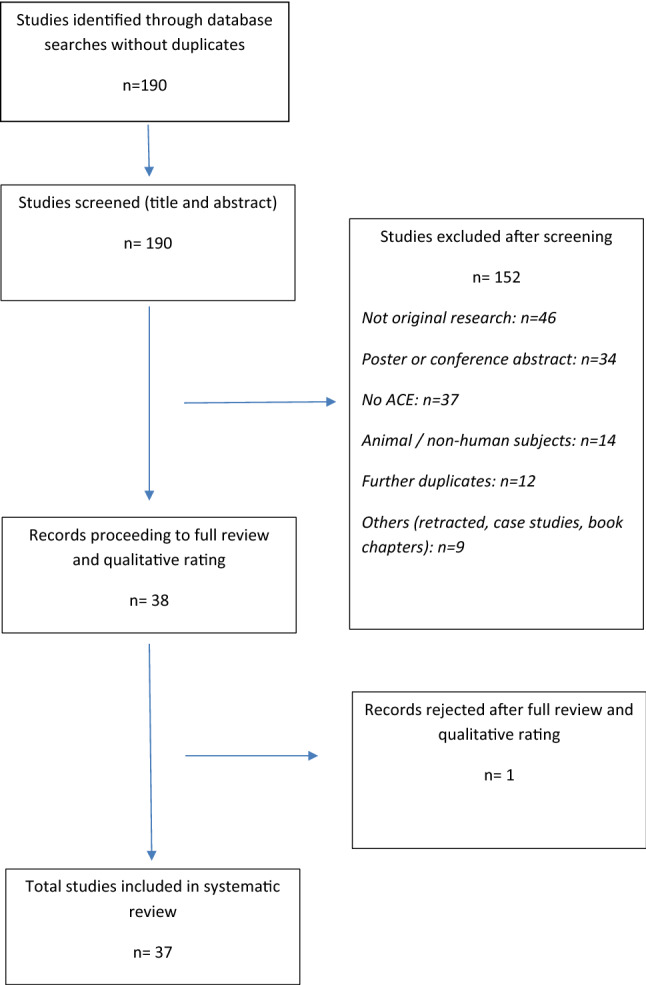
PRISMA flowchart

After applying the search protocol described above to the three databases and omitting duplicates, 190 articles were returned. Following review of title and abstract of these articles, 152 articles were excluded as they did not meet the inclusion criteria. Please see Fig. 1 for a summary. In total, 38 articles proceeded to full review, rating and data extraction. 23 papers were rated as VHQ, 12 as HQ, 2 as MQ and 1 as LQ. This LQ paper was excluded. The scores ranged from 19/40 to 39/40.

Most reported study participants were adults (*n* = 7769) while far fewer were children (*n* = 727 aged between 2 and 18 and 633 under the age of 2). Of the 37 studies, only nine included data gathered in childhood, and seven of these included a longitudinal follow-up [7, 9, 16–20]. Table 1 shows further details of these studies. The remaining studies adopted retrospective ACE reporting measures with adult participants.

**Table 1:** Summary of characteristics: studies with participants exclusively under the age of 18

References	Study size (participants and controls)	Exposure	Study design	Genome wide or gene specific	Quality	Tissue type	Direction of effect (hyper- or hypo-methylation)
Gene-specific studies							
Fumagalli et al. [16]	56 VPT infants	NICU stay	Prospective longitudinal study	Gene specific—SLC64	VHQ	Whole blood	Hyper-methylation of SLC64
Montirosso et al. [19]	78 infants (48 pre-term 30 full term)	NICU stay	Comparative prospective longitudinal study (pre-term vs full-term infants)	Gene specific—SLC6A4	VHQ	Whole blood	Hyper-methylation of SLC6A4
Romens et al. [20]	56 children (18 physical maltreatment and 38 no report)	Physical maltreatment reports from child protection services	Cross-sectional study	Gene specific—NRC31 promotor region	VHQ	Whole blood	Hyper-methylation of NRC31 promotor regions
Tyrka et al. [41]	174 children (69 documented moderate to severe maltreatment and	Moderate to severe maltreatment	Cross-sectional study	Gene specific—NR3C1 and FKBP5	HQ	Saliva	Hypo-methylation of FKBP5
Genome-wide studies							
Brody et al. [18]	399 participants (242 treatment group 157 controls)	Parental mental ill health Exposure to “harsh parenting” Treatment group exposed to SAAF programme intervention	Longitudinal follow-up of a randomised controlled trial	Genome wide (epigenetic age)	VHQ	Peripheral blood mononuclear cells	Association with parental depression and harsh parenting with increased epigenetic ageing. Association not seen in SAAF-exposed group
Cao-Lei et al. [40]	36 off-spring from ice storm-exposed mothers	Maternal pre-natal exposure to environmental stress/natural disaster	Prospective longitudinal study	Genome wide	HQ	T cells from peripheral blood Saliva	Differential methylation of various sites across genome
Telomere studies							
Provenzi et al. [7]	46 VPT infants	NICU stay	Prospective longitudinal study	Telomere length	VHQ	Whole blood	Erosion (but not statistically significant across group)
Shalev et al. [9]	236 children	Exposure to at least one violence exposure	Prospective longitudinal study	Telomere length	VHQ	Buccal swab	Accelerated erosion of telomere length in children exposed to two or more types of violence
Inflammatory markers							
Jiang et al. [39]	422 infants	Poverty, malnutrition and maternal depression	Prospective longitudinal study	Inflammatory biomarkers	HQ	Sera of whole blood	Increased inflammatory markers associated with increased adversity and decreased neurodevelopmental attainment`

The most common psychiatric conditions considered in the studies were major depressive disorder [11, 21–25] and borderline personality disorder [23, 24, 26–29], followed by post-traumatic stress disorder [8, 23, 30, 31]. Two studies considered eating disorders [26, 32]. Anxiety disorders were considered in two studies [11, 25] with attention deficit hyperactivity disorder [29], bi-polar affective disorder [29] and substance abuse [25] considered in one study each.

Studies rated as VHQ tended to demonstrate minimisation of bias in terms of careful sample recruitment, low attrition rates, clear reporting and only minor methodological difficulties. High-quality (HQ) studies were found to still be of a good standard with robust and well-reported findings; however, contained methodological issues such as not reporting in detail how the samples were recruited. Studies rated as MQ were more likely to have more substantial methodological or reporting deficits which may have been related to a lack of information on how samples, or sub samples, were recruited.

## Methodology

### VHQ and HQ studies

Of the VHQ studies, five [18, 24, 33–35] took a genome-wide approach to epigenetic analysis while eleven studies [16, 17, 19–21, 23, 25, 29, 36–38] concentrated on a specific epigenetic target. Of the studies which looked at a specific epigenetic change, all considered methylation as a mechanism for altering gene expression and all showed an association between exposure to ACE and alterations in methylation of the epigenome.

Only three HQ studies were conducted with children and, as before, these studies documented exposure to ACEs during childhood [39–41]. The remaining studies relied on retrospective reporting with adult participants. No HQ studies made pre- and post-intervention comparisons. Nine of these studies examined epigenetic changes. Of these, three used a genome-wide approach [31, 40, 42] and six focussed on specific targets [26–28, 32, 41, 43]. All nine papers had DNA methylation as the focus. Two of these papers also made comment on microRNA [42, 43] but no papers considered other epigenetic mechanisms.

A range of tissues types were used across these studies including whole blood, peripheral leukocytes, saliva/buccal samples and postmortem brain tissue.

## Findings of VHQ and HQ studies

### Epigenetic findings

Perroud and colleagues (*n* = 215) demonstrated that the severity, frequency and type of ACE exposure positively correlated with methylation of NRC31 (which codes of the glucocorticoid receptor) [23]. They found that repetition of exposure and penetrative sexual abuse correlated with a higher percentage of methylation. These findings were elaborated upon in a study by Tyrka and colleagues (*n* = 99), which demonstrated increased methylation of the NRC31 promoter in those exposed to ACEs [36] and that such methylation was likely to have physiological responses; the increased methylation profiles correlated with an attenuated cortisol response to the dexamphetamine/CRH test. This test measures participants’ reactivity of cortisol levels to the administration of dexamphetamine, with an attenuated response noted in participants with increased methylation of NRC31. However, this study utilised whole blood in a low number of participants. Controversy exists around the use of different cell lines in whole blood which are thought by some authors to potentially confound the results. (See the recent paper by Slieker and colleagues as example [44].)

Cecil and colleagues observed methylation signatures which were distinct for types of childhood abuse and also shared methylation signatures across different types of maltreatment in childhood [33]. Possible epigenetic signatures for childhood abuse and neglect, which were linked to processes involved in growth and neural development, were identified. However, the study includes low numbers of participants (*n* = 124). The findings may support a link between ACE exposure and poor physical health; however, buccal cells were used for DNA extraction which also is thought by some authors to potentially confound results [45].

Labonte and colleagues’ study (*n* = 41) suggested that both mechanisms of methylation (hypo- and hyper-methylation) are widespread and associated with ACE exposure [42]. Their examination of postmortem brain tissue demonstrated a link between methylation and transcription in neuronal tissue which could adversely affect brain function and neuronal plasticity. Again low numbers of participants is a major issue regarding the weight of the findings. Further evidence of a correlation between severity of exposure and the level of methylation detected in relation to the NRC31 gene was presented by Martin-Blanco and colleagues in a relatively large VHQ study (*n* = 281) using peripheral leucocytes [27]. This study showed a correlation between NR3C1 methylation and exposure to ACE, specifically childhood physical abuse. They linked the methylation status to the severity of symptoms of mental illness in participants with a diagnosis of borderline personality disorder.

In a VHQ study, Prados and colleagues found an association relating to microRNA 124-3 and ACE exposure; this was associated with both borderline personality diagnosis and the severity of childhood maltreatment [24]. This microRNA is associated with the expression of several genes including NR3C1, which may represent another mechanism of alteration of expression in this gene.

Perroud and colleagues’ investigated 115 participants with BPD and 52 controls pre- and post-intervention with dialectical behavioural therapy. They found a correlation between the numbers of traumatic experiences that a participant was exposed to and methylation of the brain-derived neurotrophic factor (BDNF) promoter [28]. BDNF is a gene which codes for proteins involved in the regulation, growth and plasticity of neuronal tissue. Results were mixed; with an overall increase in methylation post-intervention although the authors note this was influenced by non-responders. Additionally no correlation with blood protein levels was noted, placing in doubt the significance of the biological mechanisms involved. Methylation of BDNF loci was found to be associated with increased rates of depression, reported feelings of hopelessness and impulsivity. Although statistically significant results are reported, the number of participants was low and definitive conclusions cannot be drawn from the results. Brody and colleagues in a larger study (*n* = 399) compared epigenetic changes over time pre- and post-intervention [18], and suggested that a reduction in harsh parenting might account for protective epigenetic effects of reduced cellular ageing. These researchers seemed to demonstrate a change in methylation after exposure to psychotherapeutic treatment modalities, possibly demonstrating a biological response to psychological therapy and experience; however, replication of these findings will be required.

Mehta and colleagues examined 169 participants with PTSD; however, only 32 had PTSD and retrospectively reported ACE exposure. Methylation levels appeared to be more specific in those who developed PTSD related to childhood abuse than for those who developed this disorder secondary to other forms of trauma [31]. However, as noted, low numbers and retrospective reporting are potential significant confounders.

In a rare longitudinal study examining 36 children of survivors who were pregnant at the time of a natural disaster which caused prolonged maternal stress in Canada, Ceo-Lei and colleagues demonstrated an apparent relationship between objective maternal hardship and methylation levels associated with 957 separate genes in the offspring [40]. These changes, predominantly related to the immune system, may show the importance of foetal programming in which mechanisms of epigenetics may prepare a phenotype primed for potential hardship, although the low number of participants prevent definitive conclusions.

### Telomere studies

Telomeres were the target of eight studies in the VHQ group. Six demonstrated a shortened telomere length correlating with ACEs [8–12] and one showed a link between shortened telomeres and ACE exposure which was not sustained over time [22]. One paper showed no link between telomere length and exposure [30]. Tyrka and colleagues reported shorter telomere length in adults exposed to ACEs, even after controlling for confounders [10]. However, Verhoven and colleagues found no correlation with telomere length if the ACE had occurred more than 6 years previously [11]. These studies are confounded by methodological and life course time point differences (for discussion, see Martin Ruiz and colleagues, and Kooman and colleagues [46, 47]).

Controversy exists around the concept of whether there is a dose–response relationship between ACE exposure and shortened telomere length. Shalev and colleagues, investigating children, found that there was a relationship between exposure to two or more kinds of violence and shortened telomere length, although numbers in this subgroup were low (*n* = 39), so definitive conclusions cannot be drawn [9]. Enokido and colleagues [48] showed an association between parenting style and telomere length and also demonstrated a gender-specific response; however, this study involved healthy participants and relied on self-report.

Vincent and colleagues [12] showed that exposure to physical neglect, rather than other forms of ACE exposure, was positively associated with reduced telomere length in adulthood. This study also relied on self-report and included low numbers of participants, potentially confounding results. Verhoeven and colleagues’ (*n* = 2936) found no association between telomere length and stressful life events occurring longer than 6 years before the point of testing [11]. The recent study by Provenzi and colleagues, looking at very preterm (VPT) infants, and considering pain inflicted in the neonatal intensive care unit (NICU) as an ACE, showed increased erosion in telomere length associated with increased pain exposure [7]. Although the findings did not meet statistical significance in the group as a whole, a trend toward greater shortening was observed with increased NICU stress exposure.

Only two HQ studies focused on telomeres [8, 49]. O’Donovan and colleagues study showed a link between shortened telomere length and exposure to childhood trauma; however, number of participants was low with childhood trauma reported in only 18 participants. Mason and colleagues found little evidence of an association with childhood sexual abuse and shortened telomere length; however, again small numbers of participants meant the study lacked power [49]. It is also pertinent to note that differences in reported associations with telomere length between studies may be confounded by the fact that telomere length is a relatively weak biomarker of ageing and exhibits substantial inter-individual variation [50].

### Biomarkers

Three additional biomarkers were considered in the VHQ studies including effects on expression of Interferon Gamma and Corticotrophin Releasing Hormone [36, 51]. Two studies included functional MRI brain imaging, demonstrating a physiological effect of the observed biochemical changes [16, 51].

## Methodology findings of MQ studies

The two MQ papers [9, 17] included a genome-wide methylation-based study and a study of traditional biomarkers including telomeres, tumour necrosis factor alpha and interleukin 6. Neither paper included child participants; rather a retrospective reporting technique was adopted to quantify ACE exposure in both papers.

## Discussion

### Epigenetic targets

The collective literature generally accepts that methylation, as a mechanism for altering gene expression, occurs in various parts of the genome associated with ACE exposure. Generally, however, the extant studies suffer from low numbers of participants and a reliance on self-reported history of exposure to ACEs, meaning that definitive conclusions are not possible.

**Fig. 2:**
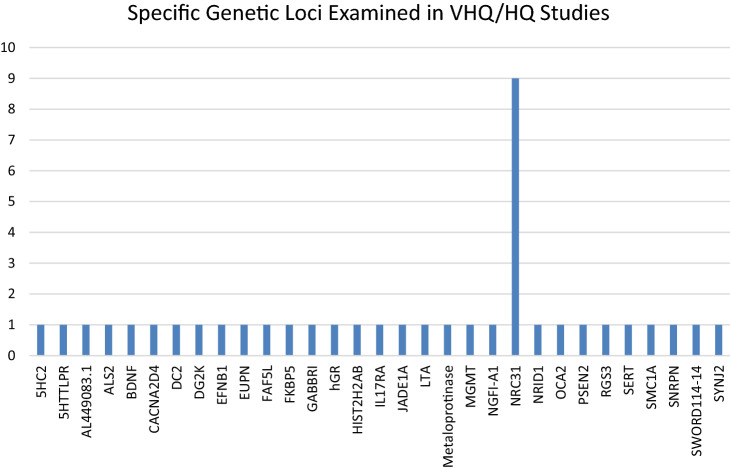
Specific genetic loci examined in VHQ/HQ studies

A diverse range of epigenetic targets were studied in the various papers selected for review, which are presented in Fig. 2. There have been a number of genome-wide methylation studies conducted which identify potential new biological pathways and targets for future research. At present, however, with a few exceptions, there is not a clear understanding of how these epigenetic changes, which seem to be associated with ACE exposure, may form part of a unified model linking past ACE exposure with current or potential future development of psychiatric disorders.

It is notable that NRC31 received most empirical attention. NRC31 codes for the glucocorticoid receptor, which binds to glucocorticoids and additionally acts as a regulator for other transcription factors. Glucocorticoids (produced in the hypothalamus) have been linked to the development and severity of several psychiatric illnesses and it is known that exposure to childhood maltreatment or adversity is associated with both changes in the hypothalamic–pituitary axis [4] and risk of developing psychiatric disorders in childhood and later years. This gene was examined in nine separate papers [20, 21, 23–25, 27, 32, 36, 52] and was noted in all nine to be epigenetically modified in association with ACE exposure. All of these studies included adult participants only.

Additionally, epigenetic targets related to the serotonin mechanism (SLC6A4, 5HT3aR, 5HTTLPR) were shown to be altered in those exposed to ACE in five papers [16, 17, 19, 29, 38].

### Non-canonical epigenetic modifications

The vast majority of studies concentrate on DNA methylation of genes or their promoters. However, there is very little evidence in the literature regarding the effect of other epigenetic modification mechanisms such as the involvement of histone modification or non-canonical epigenetic regulation by non-coding RNAs. Although this review has identified a small number of papers concerned with microRNA involvement, there is clearly a need for further studies in this area.

### Telomeres

The results of the different studies included in this review were largely in agreement, although not unanimous. There is some evidence that telomere length may be effected by exposure to ACEs, and in particular possibly more recent exposure. However, there are also a number of methodological difficulties with the included studies, and in general low numbers of participants which limit the strength of the findings. Additionally, the lack of strength of telomere length as a biomarker of ageing remains an issue [50].

### Changes in biomarkers as a result of epigenetic changes

There were few papers identified in this review which looked at changes in biomarkers related to exposure to ACE. Those papers that did involve biomarkers reported associations between ACE exposure and increased expression of IFN gamma in association with a genetic variant (rs186149) [51], increased inflammatory markers such as TNF alpha and IL 6 [39, 53] and volume reduction in the anterior temporal lobe of the brain [16]. The association with IFN gamma is intriguing, as an IFN gamma regulatory network, responsible for repression of endogenous retroviruses (e.g. LINE elements) via methylation, has recently been described as a critical mediator of biological ageing, via regulation of cellular responses to stress [54].

### Prospective studies in childhood vs retrospective studies

Although the great majority of work in this field is of high quality by virtue of robust sampling and carefully designed studies, fewer studies have examined child populations with documented cases of ACE exposure. The majority of papers characterised childhood abuse exposure using retrospective questionnaires with adults. It is likely that such recollections may have been altered over time in a way that is systematically biased according to adult outcome [55]. Retrospective studies do not allow accurate recording of the type, timing, intensity or duration of ACE exposure which may be important in identifying distinct epigenetic markers or patterns.

Prospective studies in this review did not differ in terms of their findings from retrospective studies in demonstrating possible associations between exposure to ACEs and changes in the epigenome. It could be argued that studies following children prospectively can make more detailed analysis of such changes across the life course and may be more able to identify patterns of epigenetic changes in association with known exposures [33]. Prenatal or perinatal trauma or stress, poverty or co-existing social problems are all possible confounders that can be more robustly examined using prospective designs.

### Other methodological difficulties

The field would benefit from greater consistency in methodology. For example, there are no uniform methodologies for methylation analyses in the canonical epigenetic studies. Cell types used in such studies vary, which may confound results [44] and further research investigating the relative validity of different approaches would be welcome. At the least, such variation in approach prevents direct comparison of findings between studies. Telomere-based studies also suffer from a number of drawbacks, both in methodology and in the weak nature of this biomarker [56, 57]. Also of note is that most studies, perhaps linked to availability of resources, are limited by the number of participants. This means that they will be underpowered to detect subtle changes in the epigenome in relation to ACE exposure, or may become more easily confounded by other external factors.

## Limitations

This systematic review is unique in that it has considered recent studies which characterise a range of biological changes which may occur in children exposed to ACEs in terms of inflammatory markers, structural changes, epigenetic changes and telomere involvement. However, it has a number of limitations. While efforts were made to ensure that the review was exhaustive, it is possible that other studies are available on other databases and were, therefore, not included in this review. Screening of title and abstract was conducted by only one author; however, inclusion and exclusion criteria were discussed and agreed prior to screening and where any questions arose these were discussed with other authors prior to any decision to reject. Given the variety of methodological approaches and study designs it was not possible to perform a meta-analysis of the results from the included studies.

## Conclusion

There is a considerable body of literature which supports possible epigenetic modification in those exposed to ACEs. Many studies, however, include low numbers of participants and may be confounded by other procedural factors. There is some replicated evidence for the role of epigenetic modification of NR3C1 in association with exposure to ACEs. Further work on NR3C1, especially using prospective cohorts starting in childhood, may help to elucidate the pathway(s) resulting in these changes to the HPA axis and possible later dysfunction. There has been little attention paid to other epigenetic modification mechanisms other than methylation. There remains a degree of controversy with regard to how ACE exposure affects telomere length, and this requires further clarification.

It is also likely, given the number of potential targets for future research which are identified by genome-wide studies, that future work will be able to further elucidate understanding and modelling of biological effects of other specific epigenetic targets. Various mechanisms have been postulated within the literature, and many are still to be robustly explored, as to how such epigenetic changes in ACE-exposed individuals may provide a biological basis for behaviour, personality and mental illness.

We propose that future research should be directed towards creating biological models of the impacts of ACEs with a focus on linking the various identified genes to a coherent working understanding of the role of biological mechanisms in the precipitation and perpetuation of psychiatric and physical health manifestations of ACE exposure.

Prospective longitudinal research characterising these changes as they apply to children with documented ACE exposure would strengthen the evidence base and remove potential bias in terms of subjective retrospective reporting of such experiences.
